# Germ cell and tumor associated piRNAs in the medaka and *Xiphophorus* melanoma models

**DOI:** 10.1186/s12864-016-2697-z

**Published:** 2016-05-17

**Authors:** Susanne Kneitz, Rasmi R. Mishra, Domitille Chalopin, John Postlethwait, Wesley C. Warren, Ronald B. Walter, Manfred Schartl

**Affiliations:** Physiological Chemistry I, Biocenter, University of Würzburg, Am Hubland, 97074 Würzburg, Germany; Department of Genetics, University of Georgia, Athens, USA; Institute of Neuroscience, University of Oregon, 1425 E. 13th Avenue, Eugene, OR 97403 USA; Genome Sequencing Center, Washington University School of Medicine, 4444 Forest Park Blvd., St Louis, MO 63108 USA; The Xiphophorus Genetic Stock Center, Department of Chemistry and Biochemistry, 419 Centennial Hall, Texas State University, 601 University Drive, San Marcos, TX 78666 USA; Comprehensive Cancer Center Mainfranken, University Clinic Würzburg, Josef Schneider Straße 6, D-97074 Würzburg, Germany; Texas Institute for Advanced Study and Department of Biology, Texas A&M University, College Station, Texas, 77843 USA

**Keywords:** Small RNA-sequencing, piRNA, Melanoma, Fish model

## Abstract

**Background:**

A growing number of studies report an abnormal expression of Piwi-interacting RNAs (piRNAs) and the piRNA processing enzyme Piwi in many cancers. Whether this finding is an epiphenomenon of the chaotic molecular biology of the fast dividing, neoplastically transformed cells or is functionally relevant to tumorigenesisis is difficult to discern at present. To better understand the role of piRNAs in cancer development small laboratory fish models can make a valuable contribution. However, little is known about piRNAs in somatic and neoplastic tissues of fish.

**Results:**

To identify piRNA clusters that might be involved in melanoma pathogenesis, we use several transgenic lines of medaka, and platyfish/swordtail hybrids, which develop various types of melanoma. In these tumors Piwi, is expressed at different levels, depending on tumor type. To quantify piRNA levels, whole piRNA populations of testes and melanomas of different histotypes were sequenced. Because no reference piRNA cluster set for medaka or *Xiphophorus* was yet available we developed a software pipeline to detect piRNA clusters in our samples and clusters were selected that were enriched in one or more samples. We found several loci to be overexpressed or down-regulated in different melanoma subtypes as compared to hyperpigmented skin. Furthermore, cluster analysis revealed a clear distinction between testes, low-grade and high-grade malignant melanoma in medaka.

**Conclusions:**

Our data imply that dysregulation of piRNA expression may be associated with development of melanoma. Our results also reinforce the importance of fish as a suitable model system to study the role of piRNAs in tumorigenesis.

**Electronic supplementary material:**

The online version of this article (doi:10.1186/s12864-016-2697-z) contains supplementary material, which is available to authorized users.

## Background

Small-noncoding-RNA guided gene regulation is a well-established and important branch of gene regulation. With the advent of high throughput sequencing coupled with functional studies a variety of small noncoding RNAs has been identified including PIWI-interacting RNAs (piRNAs). piRNAs interact with Piwi-family proteins and are processed by a Dicer-independent mechanism [1]. They are predominantly expressed in germline cells where they mainly act to silence transposable elements (TEs) [2]. By guiding Piwi proteins to complementary target sequences for cleavage piRNAs help to maintain genome integrity and their function has been well conserved throughout the animal tree of life [3–6]. A role of piRNAs in the conservation of the germ cell epigenomes has been postulated [7], further, evidence suggests that piRNAs play a role in stem-cell function, whole-body regeneration and cancer [8, 9]. piRNAs are generated from long precursor RNAs so called clusters, which can be up to 100 kb long, they are strongly enriched in repetitive sequences and normally encompass multiple transposon sequences [10, 11]. Biogenesis of piRNA occurs by two highly conserved pathways; primary processing and secondary pathways. [1]. During primary biogenesis piRNA clusters are transcribed and loaded onto the Argonaute family protein PIWI to be further processed into primary piRNAs. Other proteins that are involved in primary piRNA biogenesis in *D. melanogaster* are Tudor-domain-containing proteins, which directly [12, 13] interact with PIWI, which is necessary for the assembly of other proteins essential for the PIWI pathway [14]. In the secondary pathway, specific piRNAs targeting TEs are amplified in a loop, known as the “ping-pong cycle” [15, 16]. In contrast to other RNAs, piRNAs contain a 2’-O-methylated 3’ terminus which protects them from degradation, e.g. by NaIO_4_-mediated oxidation [17]. A systematic comparative analysis on different teleost fish genomes suggests that the piRNA biogenesis pathway is likely to be involved in the adaptation to transposon diversity [5]. In particular, fish genomes show a much greater diversity of transposable elements than other vertebrates [18].

**Fig. 1:**
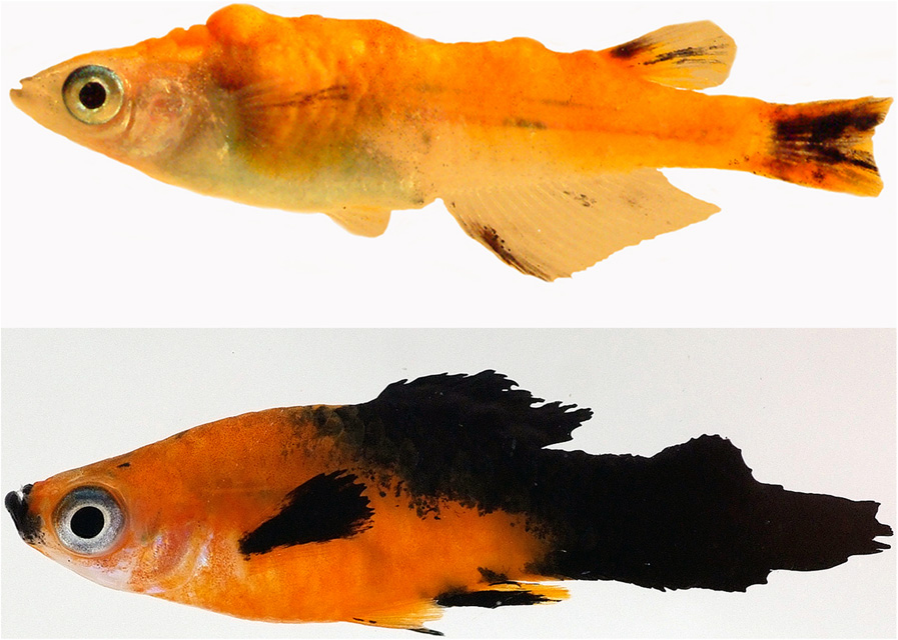
Pigment cell tumor developing fish as used in this study. Upper: mitf:xmrk transgenic medaka with exophytic xanthoerythrophoroma (XE), lower: Xiphophorus hybrid with malignant melanoma

In addition to their function in germline cells, it is emerging that piRNAs might also play a role in various somatic cell cancers. Recent studies have clearly demonstrated aberrant expression of PIWI proteins and piRNAs in variety of cancers [9, 19–22]. However, almost nothing is known about a role of piRNAs in the development of melanoma. Small laboratory fish are generally accepted and increasingly used models for a better understanding of the molecular basis of melanoma formation [23–25]. They also provide many experimental advantages for high throughput drug screening and detection of novel melanoma molecules and tumor markers. We use a natural, so-called evolutionary model of spontaneous melanoma formation in hybrids of platyfish (*Xiphophorus maculatus*) and swordtails (*X. hellerii*) (as reviewed in [26, 27]) and a transgenic model in medaka (*Oryzias latipes*), where fish expressing the *xmrk* oncogene from platyfish under the pigment cell specific *mitf* promoter of medaka develop various types of melanoma [28] (Fig. 1). The pigment cell tumors of both models have previously been shown to be comparable to human melanoma on the levels of proteins, mRNAs and they share many features with these malignancies [25, 29, 30]. In this study, we sequenced small RNAs of testes, ovary, tumor and benign control samples to investigate the role of piRNAs in different melanoma entities of our fish models.

## Results

### Construction of a piRNA cluster reference set for Xiphophorus and medaka

**Fig. 2:**
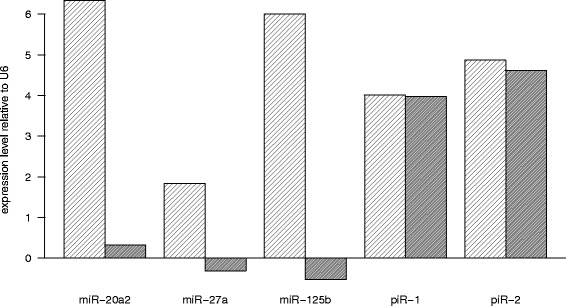
Abundance of RNA before oxidation (oxi-) and after oxidation (oxi+) of smallRNA relative U6 as control, miR-20a2, miR27a, miR-125 and two piRNAs, tested by qPCR. After oxidation a strong reduction of miRNAs, but no decrease of the amount of piRNAs can be observed, confirming the efficacy of the oxidation procedure

So far, piRNA reference data only exist for a few organisms. Because piRNAs are very poorly conserved, we first had to construct a reference dataset for *Xiphophorus* and medaka (Additional file 1: Figure S1). Therefore, we mapped sequences of oxidized small RNA samples of testes of both fish, which should contain only piRNAs protected by 3’ end 2’-O-methylation, to the respective genomes and the hits were merged (see Material and Methods). Sequencing with the Illumina HighSeq™ system produced 10^6^ clean reads for oxidized samples of medaka and *Xiphophorus* (Additional file 2: Table 1). Percentage of clean sequences was between 98.73 and 99.67 % of the total reads. In medaka 28 % and in *Xiphophorus* 39 % of the piRNAs were sequenced with fewer than 10 reads and these extremely low expressed sequences were removed. To confirm the efficacy of the oxidation procedure, two putative piRNA cluster loci, U6 RNA and the miRNAs miR-20a2, miR-27a, miR-125 were tested by qPCR, comparing RNA from the samples before and after oxidation. U6 and all miRNAs showed a strong reduction in abundance. In contrast, both piRNA clusters showed almost no change (Fig. 2), indicating that only piRNA was protected from degradation during oxidation. This conclusion was supported by the length distribution of the sequences remaining after oxidation with a clear peak at 28 nt (Additional file 3: Figure S2). To obtain a preliminary reference oxidized testis samples were mapped to the respective genome and the hits were merged. With a spacing of 1 kb this procedure resulted in 175698 unique clusters for medaka and 114741 unique clusters for *Xiphophorus*. To reduce the risk of contamination with remnants of other RNAs, which may be present in somatic tissues as well as in germline cells, the non-oxidized samples were filtered (see methods) and then mapped to the preliminary reference. After excluding unreliable clusters the final reference consisted of 110263 separate clusters with an average length of 2099 nt for medaka and 45461 separate clusters with an average length of 579 nt for *Xiphophorus*. This high number of clusters resulted from the extremely small spacing of 1 kb that we allowed between two consecutive piRNAs.

To verify that no microRNAs contaminate our reference datasets, we blasted the reference sequences to known microRNAs from mirBase. No regions overlapping miRNAs were detected in either species. In contrast, 43.7 % of the medaka reference sequences had *Blast* hits to known fish TEs (% identity > 90 %). Of the *Xiphophorus* reference, 70.2 % of the sequences had *Blast* hits to known fish TEs. Most piRNAs were present in ovary of both medaka and *Xiphophorus*, but at lower levels than in testes, like in zebrafish [31].

### Transposable elements (TEs) in medaka and Xiphophorus

**Fig. 3:**
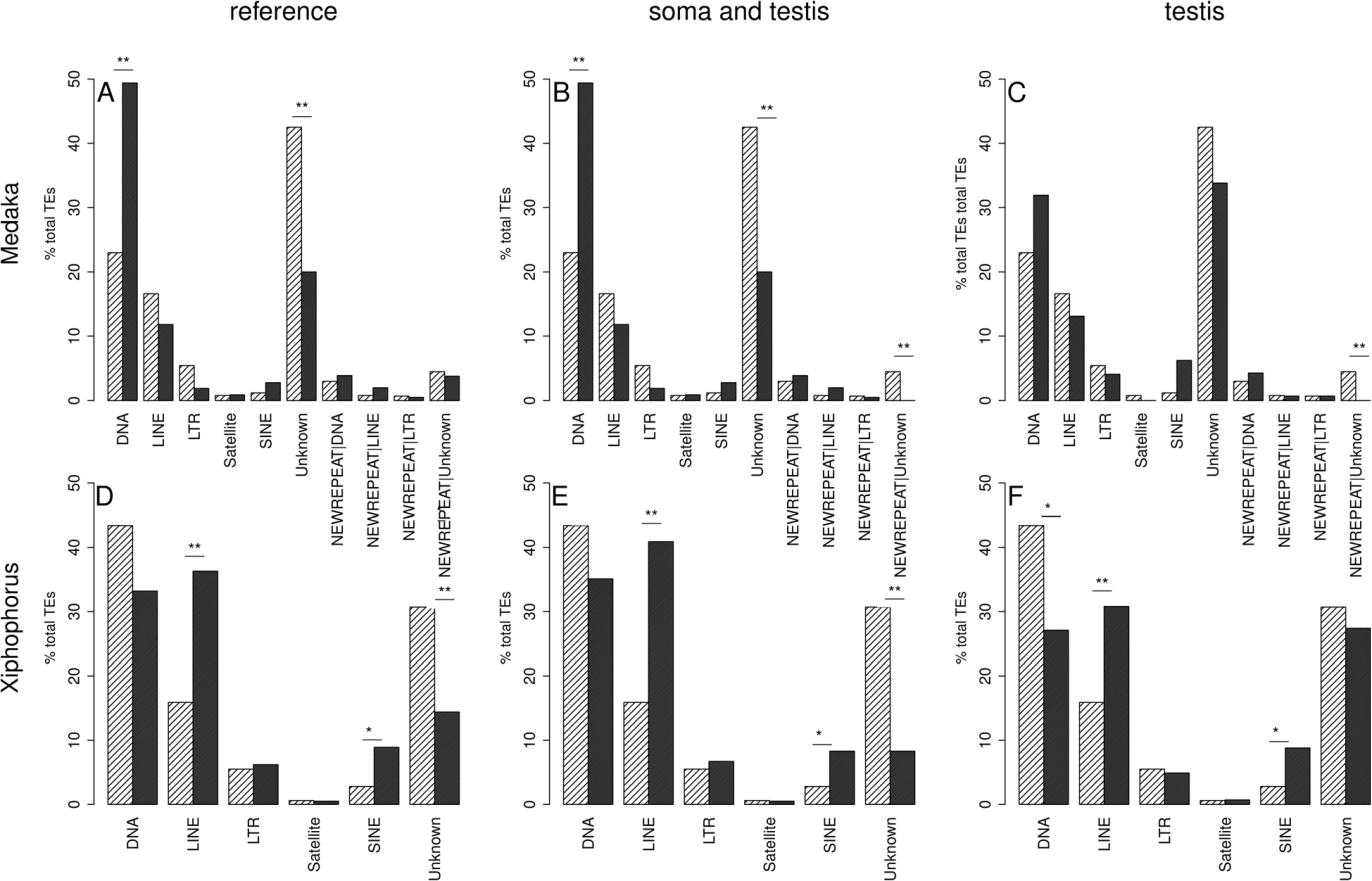
Barplot of proportions of TE classes present in medaka (**a**-**c**) or *Xiphophorus* (**d**-**f**)*. Medaka:* piRNA reference set (**a**, *dark bars*), in both somatic tissue and testis (**b**, *dark bars*) and in testis only (**c**, *dark bars*) in comparison to all known medaka TEs (*light bars*). DNA TEs are significantly up-regulated in the reference and in piRNA clusters that are present in both somatic cells and testis, whereas the group comprising unknown TEs is significantly down-regulated in the soma and testis and testis only. Xiphophorus: piRNA reference set (**d**, dark bars), in both somatic tissue and testis (***e***, *dark bars*) and in testis only (**f**, *dark bars*) in comparison to all known Xiphophorus TEs (*light bars*). LINEs and SINEs are significantly up-regulated in all data sets, whereas the group comprising DNA TEs is significantly down-regulated in testis. Unknown TEs are significantly down-regulated in the reference set and in the group of piRNA clusters present in soma and testis. * *p*.value <0.1, ** *p*.value < 0.05

Our TE library contained 1012 TEs for Xiphophorus and 994 TEs for medaka. Out of these, 590 TEs had a Blast hit on the *Xiphophorus* piRNA reference and 716 TEs had a Blast hit on the medaka piRNA reference. Comparing the proportions of TE classes of all known TEs with the TE classes with Blast hits on the piRNA reference we found in *Xiphophorus* an enrichment of LINE and SINE elements. The number of unknown TEs was reduced primarily in piRNA clusters present in both testis and somatic cells (*p*.value < 0.01) (Fig. 3 d-f), the number of piRNA sequences with similarity to DNA TEs was reduced in piRNA clusters found in testis only. In medaka, however, there was a significant enrichment of piRNA clusters with Blast hits to DNA TEs and, like in Xiphophorus, a reduction of unknown TEs in somatic cells (*p*.value < 0.01) (Fig. 3 a-c). Of note, there are about twice as many DNA TEs known in Xiphophorus than in medaka.

### Differing base preference at the first position of piRNAs between germline cells and somatic cells

Due to processing, piRNA sequences have a preference of uridine at the first position and adenine at the 10th position [32]. Looking at the base distribution of the oxidized small RNAs with hits on the references, we found a significant bias towards uridine at the first position (A: 10.8 %, C: 6.1 %, G: 5.9 %, U: 77.2 % for medaka and A: 1.3 %, C: 1.4 %, G: 2.3 %, U: 95.0 % for *Xiphophorus*) and adenine at the 10th position (A: 49.1 %, C: 13.1 %, G: 18.0 %, U: 19.8 %) in medaka (Chi-square test, *p*.value < 0.01), which is in in agreement with other studies [12, 33]. Base distribution at the first position of putative piRNAs from ovary and testis was similar to the oxidized samples from both fish species (Additional file 4: Figure S3 A and C). Also healthy skin from fins was more similar to the oxidized RNA from testis than to the control sample. To define a preference for either the primary or secondary processing pathway of piRNAs we calculated the primary/secondary pathway ratio as described in Aravin et al. [32]. According to the base preference during piRNA processing, piRNAs derived from the primary pathway are defined as having uridine at position 1 but no adenine at position 10 (10A). piRNAs processed in the secondary pathway are defined as having any base but uridine at position 1 and adenine at position 10. In addition, because some of the samples showed a clear preference for guanine at position 1 or 10, we calculated a ratio with 10G in the same way. All other sequences, were excluded. Ratios 1U/10A were significantly higher in germline cells, indicating that there is a higher proportion of piRNAs processed in the secondary pathway in the somatic cells than in germline cells. This bias was even more obvious for 1U/10G (Additional file 5: Table S2).

In medaka (Additional file 4: Figure S3 A and B) less aggressive tumor samples had a bias towards guanine at the first position in piRNA sequences. Tumors and HP did not have a large bias. Only UM and IM, which are the most aggressive melanoma types, showed a bias to uridine at the first position (1U), albeit not as clearly as in germ line cells. In *Xiphophorus* piRNAs at position 10 had a bias to guanine at position 10 (10G) in all samples except ovary (Additional file 4: Figure S3 A-D).

### PIWI and tudor proteins are expressed in medaka melanomas

**Fig. 4:**
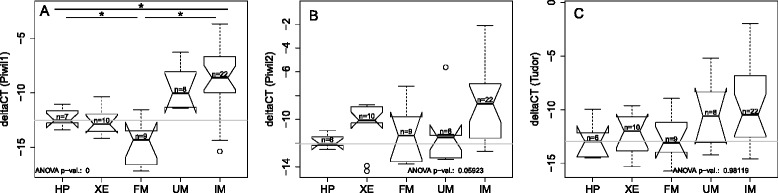
Boxplots of the abundance of piwil1 **a**, piwil2 **b** and tudor **c** measured by qPCR. In comparison to HP Piwil1 and Tudor are enriched in uveal melanoma (UM) and invasive melanoma (IM). Piwil2 is enriched in xanthoerythrophoroma (XE) and IM. All values are expressed relative to ef1a as reference

To investigate, how somatic tissues of medaka express Piwil1, Piwil2 and Tudor, the enzymes involved in the primary processing pathway of piRNAs, we determined the expression levels in HP and different types of pigment cell lesions and tumors by qPCR (Fig. 4). In comparison to HP piwil1 was significantly upregulated in IM, which is a more aggressive form of skin cancer. In less malignant melanoma subtypes piwil1 showed only low levels of expression (Fig. 4a). In contrast, piwil2 was up-regulated in XE and IM with the highest median expression levels in IM (Fig. 4 b). Tudor tends to be upregulated in XE, FM and IM (Fig. 4 c).

### Expression of piRNA clusters in melanoma

Having shown that pigment cell tumors indeed express piwil1, piwil2 and tudor, the next step was to look for the presence of piRNA in these samples. Therefore, we sequenced the small RNA fraction of several melanoma types, premalignant lesions and testes and ovaries. Analysis resulted in approx. 2.5*10^7^ clean reads for somatic tissue samples and 3*10^7^. clean reads for testis and ovary. Subsequently, small RNA sequences were filtered (see methods) and mapped to the respective final reference. All sequences that did not map to the reference were excluded.

### Medaka

**Fig. 5:**
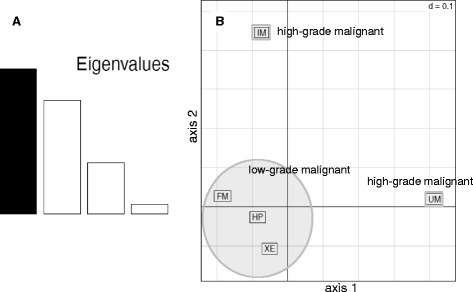
Correspondence analysis (COA) of tumor and control samples of medaka. **a** Associated Eigenvalues, with the relative high second bar indicating that there is still meaningful information in the second component (axis2). **b** On axis1, less aggressive lesions, xanthoerythrophoroma (XE), fin melanoma (FM) and hyperpigmented skin (HP) are clustered together and are clearly separated from uveal melanoma (UM), which is a high-grade malignant melanoma. Invasive melanoma (IM) is separated by the second component from all other samples

**Fig. 6:**
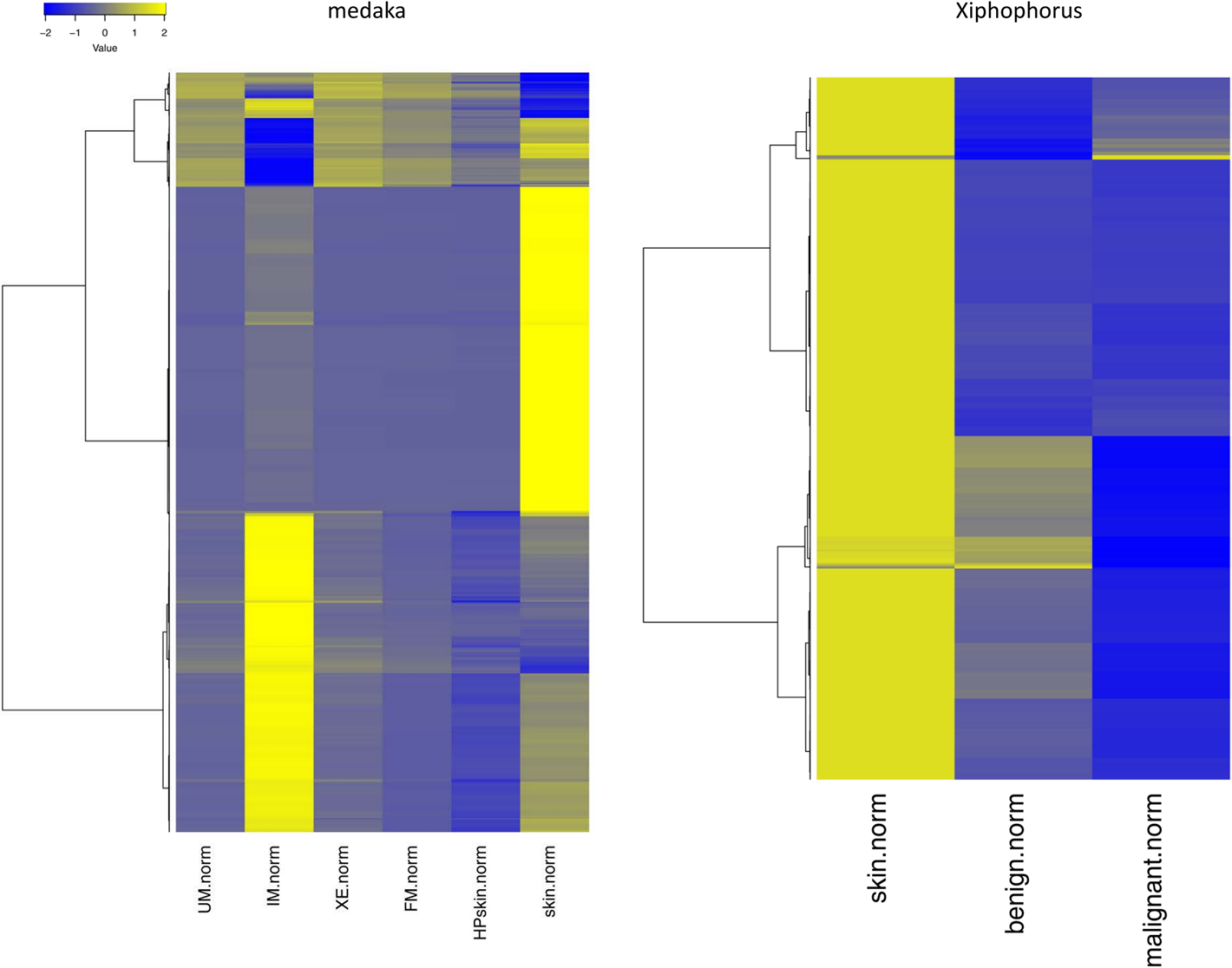
Row wise scaled heatplot of normalized expression levels of piRNA clusters: Diffenertially expressed piRNA clusters with fold change > 4-fold and *p*.value < 0.05 between tumor and HP in medaka or malignant and benign in *Xiphophorus*. In addition the corresponding expression levels of normal skin is shown. piRNA clusters are both up and down regulated. In both fish there is a large amount of clusters showing a much higher expression level in skin than in any other somatic tissue measured

In total we found 9006 clusters that were expressed in testis only. 70582 clusters were expressed in tumor samples and hyperpigmented skin as the non-malignant control. Comparing the expression of malignant versus benign control tissue, in medaka 2098 (3.0 %) of the clusters were down-regulated more than 4-fold in IM as compared to HP and 1140 (1.6 %) were up-regulated. Correspondence analysis (COA) clearly distinguished between non-malignant and malignant samples (Fig. 5). In heatmaps of piRNA clusters with a fold change > 4 and a *p*.value < 0.05 for the comparison of HP vs. IM, most of the clusters were up-regulated in IM. However, there was a group of clusters which was also up-regulated in IM but even higher in normal skin (Fig. 6).

### Xiphophorus

Calculating the distribution of clusters within samples showed that *Xiphophorus* had 7245 clusters that were expressed exclusively in testis. Differential expression between tumor samples and hyperpigmented skin as the non-malignant reference was calculated for the piRNA clusters expressed in both testis and somatic cells (*n* = 32694). Comparing the expression of malignant versus benign control tissue, 371 (1.1 %) of the clusters were down-regulated more than 4-fold but only 38 (0.1 %) of the clusters were up-regulated in the malignant tissue samples. In *Xiphophorus* almost all clusters that were differentially regulated between benign and malignant tissue had higher expression levels in skin than in other somatic tissues and thus show more similarity to germline cells (Fig. 6).

### Confirmation of piRNA regulation and detection of putative target in medaka

**Fig. 7:**
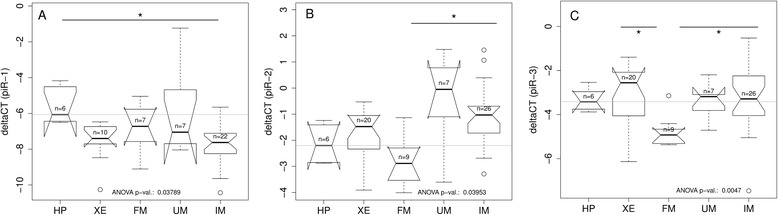
Boxplots of the abundance relative to U6 for selected piRNAs (piR-1: **a**, piR-2: **b**, piR-3: **c**). The grey line indicating the median HP value

**Fig. 8:**
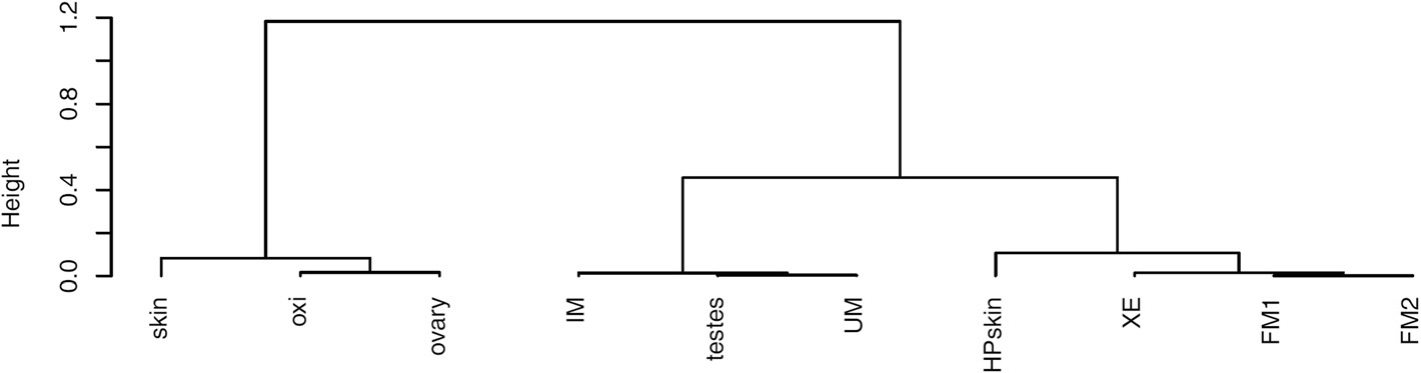
Dendrogram, based on the abundance relative to U6 of the selected piRNAs. Using only these three piRNAs it is possible to separate low malignant tumors (XE, FM) and HP from high malignant tumors (IM, UM) and testes. Skin, oxidized testis and ovary are clustered in one distinctly separated group

To confirm the differential expression of piRNA clusters on a larger melanoma sample set, we selected three piRNAs and validated their expression by qPCR (Fig. 7). piRNA1 was slightly down-regulated in all tumors. piRNA2 was up-regulated in FM, UM and IM in comparison to HP and piRNA3 was down-regulated in FM. Based on qPCR results of these three piRNAs it is possible to differentiate between low-grade malignant tumors (FM, XE) and HP and high-grade malignant tumors (IM, UM) which cluster with piRNAs from testes (Fig. 8). Next, putative target genes were determined using RNAhybrid. Targets for piRNA1-3 are listed in Additional file 6: Table S3. It is noticeable that, when comparing these putative targets with the gene expression data of an earlier study [30] a significantly (2-sample test for equality of proportions *p*-value < 0.001) higher percentage was down-regulated than up-regulated more than 2-fold (mean_down_: 46 %, mean_up_: 10 %). In contrast, genes with a maximal distance of 10 kb to piRNA clusters tend to be more up-regulated fold (mean_down_: 19 %, mean_up_: 32 %). In the complete dataset about the same number of genes was up- regulated and down-regulated. Functional analysis of the targets using DAVID revealed many genes involved in purine ribonucleotide and ATP binding.

### Comparison of piRNAs between medaka and Xiphophorus

In medaka 20 % of the reference piRNA sequences mapped to 9632 unique genes, while in *Xiphophorus* only 2 % of the reference piRNA sequences mapped to 1615 unique genes, 604 of these genes were found in both fish. In this common gene set pathways involving among others apoptosis and ‘Glycine, serine and threonine metabolism’ were enriched. Gene ontology (GO) analysis also revealed an enrichment of genes related to the categories ‘response to endoplasmic stress’and ‘nucleobase biosynthetic process’ (Additional file 7: Figure S4). Comparing *Xiphophorus* and medaka reference clusters by *Blast* (%identity > 90 and score > 1000) resulted in 425 hits with an alignment length between 693 to 1645 bases. Interestingly, there was a discrepancy between the number of unique hits for *Xiphophorus* (24 unique clusters, mean cluster length 2341 bases) and medaka (255 unique clusters, mean cluster length 5147 bases). Thus, many clusters in *Xiphophorus* had various matching members in medaka, but only a few medaka clusters corresponded to more than one sequence in platyfish. These sequences did not overlap with human sequences, yet, comparing gene names resulted in an overlap of 150 genes (Additional file 8: Figure S5), which were significantly enriched in purine ribonucleotide and ATP binding.

## Discussion

In this study we explored piRNAs of medaka and *Xiphophorus* and found different expression patterns in germline cells, different melanoma types and healthy skin.

Prior to developing our own piRNA clustering pipeline we tested two already publicly available tools, proTRAC [34] and piClust [35]. We excluded piClust, because of the restriction to selected genomes, file size and the limited number of multiple mapping sites per read. Using our dataset proTRAC resulted only in a very small number of results, most likely because proTRAC includes typical piRNA and piRNA cluster characteristics such as the number of loci with a T at position 1 or A at position 10. Even though Piwi-like and Argonaute-like protein 3 (Ago3) are present in both fish, Ago3 is not well conserved, so we did not want to assume a certain nucleotide distribution. Because we are working with fish genera in which piRNAs have not been studied before, we wanted to be sure that presumptions based on other model organisms did not lead to false negative results and therefore decided to develop our own pipeline. To our knowledge, no commonly accepted rules yet exist to computationally define piRNA clusters in fish. Additionally, the number of clusters found in different organisms is extremely variable, likely due to the heterogeneity of individual sequences and the poor conservation of clusters among species. For example the piRNA bank (http://pirnabank.ibab.ac.in) contains 114 entries for human, 2710 for mouse and 7094 cluster entries for zebrafish. Due to this disparity piRNAs from other eukaryotes sequences could not serve as a guideline. To overcome these problems, we oxidized and subsequently sequenced small RNA from testes of both fish. We verified that oxidation is indeed an efficient method to enrich small RNAs preparations for piRNAs and exclude other small RNA types. In addition, to eliminate remainders of any other types of RNA and piRNA clusters expressed at very low levels, sequences were prefiltered based on known datasets for ncRNA and for simple repeats. Because we focused on the role of piRNAs in the development of melanoma, we chose an extremely short distance between two piRNA sequences to be sure to still have a good resolution to find differentially expressed clusters. On the other hand we did not want to keep a large number of redundant separate sequences and so we condensed data as much as possible without introducing errors from over condensing. By finding 110262 clusters on 6330 different genome scaffolds for medaka and 45461 clusters on 10044 genomic scaffolds for *Xiphophorus*, our reference sets contain many more clusters than have been found in for example zebrafish. However, with greater spacing differences in piRNA cluster expression intensity tend to be underestimated and expression differences of smaller piRNA sequence might be averaged. Previous attempts with a larger spacing showed a too low resolution to reliably find differentially expressed piRNA clusters (data not shown). Our data showed that highly expressed piRNAs tend to be clustered. Changing the criterion for defining a cluster from 1 kb to 10 kb would result in 11173 clusters for medaka and 30473 clusters for *Xiphophorus*. The high number of clusters for *Xiphophorus* with a 10 kb spacing can be caused by an almost 3x higher number of genomic scaffolds and a 4.6x shorter scaffold N50 in the *Xiphophorus* genome compared to medaka.

Regarding the base distribution of sample sequences, medaka has a bias toward 1U and 10A in the sequences of germline cells as described by other studies [3, 33]. Our sequence data showed that somatic cells of medaka have a bias towards guanine at position 1 and toward uridine at position 10. This bias might partly be explained by the preferential usage of either Piwil1 or Piwil2 in these tumor types as indicated by qPCR (Fig. 4). Further, calculating the ratio 1U/10A as a measure for the preference of one or the other biogenesis pathways [36] indicates that the secondary pathway generates most piRNAs in somatic cells. This apparent pathway bias is in line with the higher number of piRNAs expressed in somatic cells than piRNAs expressed in testis only (Table 1).

**Table 1:** Percentage of sequences mapping to a known transposable element (TE)

	Testis	Somatic
medaka	15.30 %	60.30 %
*Xiphophorus*	72.60 %	73.30 %

Direct comparison of medaka and *Xiphophorus* piRNA clusters showed that more *Xiphophorus* clusters had positive *Blast* hits to medaka than vice versa. These and other features that are different between the piRNA dataset from the two melanoma fish models may be due to the fact that the medaka melanoma model is a transgenic model where tumors develop on a purebred, rather homogenous wildtype background, while in the *Xiphophorus* model, the tumors develop on an interspecific hybrid genetic background. In several studies piRNA origin has been linked to transposons [2, 37]. In a hybrid genome TEs can be activated [38] and influence piRNA clusters. It has been shown that there is a great variability of TE content and type in different fish [17], which can explain the diversity of the piRNA cluster sequences in our two models.

To compare genes overlapping with fish and human piRNA clusters, human piRNA sequences we also merged with an inter-piRNA maximum distance of 1 kb, which resulted in 900 clusters. This increase in piRNA clusters from 114 to 900 corresponds to about the same rate as for medaka. Comparable to the results in other organisms [39], we found only poor conservation between *Xiphophorus* and medaka reference sequences and none of these cluster sequences were found to be conserved in human [37].: The 150 fish genes with overlap to piRNA clusters that are also found in the human genome may be underestimated, because the list is limited to known homologs from the Ensembl database. In our list 79 % of Xiphophorus protein-coding genes and 74 % of medaka protein-coding genes have human homologs.

We decided to use hyperpigmented skin in medaka and benign melanoma in *Xiphophorus*, which is comparable to HP, as controls, because healthy skin as control did not seem to be useful for normalization, because samples of healthy skin from caudal fins in medaka and dorsal fins in *Xiphophorus* tended to show an expression pattern with higher piRNA levels, somewhat more similar to germline cells (Fig. 6). This is consistent with a previous study, where piRNA from embryonic stem cells (ESC) and human skin had higher expression levels than samples from human saliva [40].

Our dataset showed overexpression of *piwi* genes, and the same result is frequently found in human cancers [41], The murine PIWI/AGO gene subfamily MILI has been found to be able to methylate LINE1, which is crucial for the expression of melanoma antigen family A (MAGEA) [42] and thus for tumor progression. We found that some piRNA clusters are down-regulated and others are up-regulated in tumor cells. Both up-regulation and down-regulation of piRNA biogenesis has been linked previously to several cancers such as breast cancer [21, 43], pancreatic cancer [44] or bladder cancer [45]. Down-regulation of human piRNA-823 has been observed to promote gastric cancer [46]. Furthermore, piRNA expression can not only distinguish between tumors and non-malignant tissue, but also delineate clinical features, such as histological subgroups, disease stages and survival [47]. PIWIL1,2,3 and PIWIL4 have been found to be mutated in skin cancer [22]. Human piRNA_015520 was shown to negatively regulate the human melatonin receptor 1A gene, which is expressed in adult human testes and brain [48]. A possible role of piRNAs and PIWI proteins as diagnostic and prognostic biomarkers has been discussed [49].

## Conclusions

To our knowledge, this is the first comprehensive study of piRNAs in melanoma at all. In previous studies we showed that in Xiphophorus TX-1, an active LTR-containing retrotransposon, causes a disruption of the Xmrk oncogene and thus repressed tumor formation [50]. A causal relationship between TEs and cancer has also been discussed by others [40, 41, 51, 52]. Our results suggest that certain piRNAs are differentially regulated in more aggressive melanoma subtypes compared to hyperpigmented skin. Functional studies in fish melanoma cell lines, by modulating piRNA levels and observing phenotypic changes will have to be conducted to further elucidate the role of piRNAs in melanomagenesis and can be followed up by functional studies in-vivo including manipulation of expression of piRNA biogenesis proteins and levels of selected piRNAs in fish melanoma.

## Methods

### Experimental animal and sample collection

All animal studies were approved by the Institutional Review Board (Animal Welfare Officer of the University of Würzburg). All fish used in this study were from aquaria housed stock and were kept and sampled in accordance with the applicable EU and national German legislation governing animal experimentation. We hold an authorization (568/300-1870/13) of the Veterinary Office of the District Government of Lower Franconia, Germany, in accordance with the German Animal Protection Law (TierSchG).

The transgenic medaka melanoma model was obtained by stable expression of the melanoma oncogene *xmrk* from *Xiphophorus* under control of the pigment cell specific medaka *mitf* promoter [28]. Spontaneous development of melanoma in *Xiphophorus* is achieved by inter-specific crossing of female *X. maculatus* and male *X. hellerii* and then backcrossing of F1 hybrid females with wild type male *X. hellerii* (classical or Gordon-Kosswig-Anders cross) as described previously [26]. The different types of melanoma were generated as described previously [53]. The details of the fish genotypes and the respective category of pigment cell lesions are described in Table 2. For dissecting normal organs and tumor samples fish were sacrificed by over-anesthisation with Tricaine.

**Table 2:** Samples used for small RNA sequencing

	Pool of
Medaka sample	
Testis oxidized	10
Testis	10
Ovary	10
Uveal melanoma (UM)	2
Invasive melanoma (IM)	2
Exophytically growing xanthoerythrophoroma (XE)	4
Fin melanoma (FM1)	3
Fin melanoma (FM2)	3
Hyperpigmented skin (HP)	10
Skin	6
*Xiphophorus* sample	
Testis oxidized	1
Testis	1
Ovary	1
Control (dorsal fin)	5
Benign melanoma	2
Malignant melanoma	1

### *RNA isolation and* oxidation

RNA was extracted from pooled testis, ovary, normal skin, hyper-pigmented skin (HP), benign premalignant lesions, and different tumor tissues from medaka (Ol), *Xiphophorus hellerii* (Xhe) and *Xiphophorus* backcross hybrids. Small RNAs (<200 nt) were isolated with the RNeasy MinElute Cleanup kit (Qiagen, Germany) from medaka testis, ovary, skin, hyperpigmented skin, fin melanoma (FM1, FM2), xanthoerythrophoroma (XE), uveal melanoma (UM) and invasive muscle melanoma (IM) and *Xiphophorus* testis, benign melanoma and malignant melanoma. In medaka and *Xiphophorus* piRNAs sequences have not been described prior to our study. To identify piRNAs in the small RNA fraction we made use of the fact that the 3’ ends of piRNAs are known to be resistant to oxidation because of 2’-O-methylation (Zamore Lab Illumina TruSeq Small RNA Cloning Protocol (April, 2014)). To enrich for 3’-end modified small RNAs and also to establish a piRNAs reference dataset, small RNAs (<200 nt) from medaka testis and *Xiphophorus* testis were oxidized by treating with NaIO_4_. Briefly, the small RNA fraction was incubated for 30 min with 25 mM of freshly prepared NaIO_4_ in borate buffer (50 mM sodium tetraborate decahydrate and 50 mM boric acid; pH 8.6) in a final volume of 40 μl. Then 30 μl of 3 M sodium acetate (pH 5.2) and 1 μl glycogen was added. RNAs were precipitated at −70 °C for 1 h, and then the precipitate was collected by centrifugation and redissolved in an appropriate volume of RNAse-free water [50]. All small RNA samples were then size selected (<35 nt) and messenger RNAs and small RNAs were custom sequenced on an Illumina platform by BGI-tech (Shenzen, China).

### General prefiltering steps

A flow chart of the reference construction is shown in Additional file 1: Figure S1.

Several filtering steps were applied to the sequencing files of all samples before further processing. Adaptors, low quality tags as well as contaminants were removed with inhouse software by the BGI (http://bgi-international.com/services/bioinformatics-analysis/). To exclude microRNAs from the analysis, only sequences between 25 and 32 nucleotides were selected and sequences with hits on known miRNAs from mirbase (hairpin, version 20) were omitted. For medaka, tRNA was eliminated based on known medaka tRNAs from the genomic tRNA database (http://gtrnadb.ucsc.edu/, *Oryzias latipes*, Oct 2005). Based on the *.out file resulting from repeatmasker (http://www.repeatmasker.org) remaining tRNAs, rRNAs and simple repeats were excluded. All other ncRNAs like long terminal repeats (LTRs) were retained. For read mapping the SeqMap tool, which is designed to map short sequences to the genome, was used with the argument ‘output all matches’ with no mismatches allowed [51].

### Construction of a pre-reference data set for Xiphophorus and Medaka

So far, no piRNA reference dataset for medaka or *Xiphophorus* is available. To construct a reference, sequences of the oxidized testis samples were preselected as described above and mapped to the medaka genome (Oryzias_latipes.MEDAKA1.74.dna.toplevel) and the *Xiphophorus* reference genome (*Xiphophorus*_maculatus.Xipmac4.4.2.76.dna.toplevel), respectively. To detect piRNAs that are clustered on a closely spaced genomic region, chromosomal location and strand of the sequences were extracted to build a BED file. Using mergeBed (BEDTools) [52], sequences lying within an interval of up to 1 kb (option –d 1000) were merged into a common cluster. We decided to choose a relatively short [36, 54–56] maximal distance between piRNA sequences to achieve a higher resolution for the detection of differentially expressed clusters in our comparisons of normal and tumor samples. Sequences of these clusters were used as a preliminary piRNA reference set. In further analyses sample sequences mapping to these regions were considered to be putative piRNAs.

### Detection of piRNAs in melanoma and construction of the final reference

In the next step the remaining samples (Table 2) were mapped to these newly established preliminary reference datasets using segmap (option: /output_all_matches, no mismatch). These samples were not oxidized but their sequences were filtered as described above. To get a final reference set, unexpressed or questionable clusters were excluded as follows: clusters with a read count < 10 for both the oxidized sample and the unoxidized testes sample. Further, to get the final references, clusters with a ratio of oxidized/non-oxidized testis sample of < 0.1 were excluded supposing these sequences to be remainders of extremely highly abundant miscellaneous RNAs in the testis.

To test whether there is a difference between piRNAs being expressed in testes only and piRNAs that are differentially expressed between melanoma and HP, two piRNA sets were selected for each species of fish. The first set contained piRNA-clusters showing expression in testes primarily and was defined – with the threshold for sum(read count) equal to the number of samples - as:

$$ \mathrm{sum}{\left(\mathrm{read}\ \mathrm{coun}\mathrm{t}\right)}_{\mathrm{somatic}} < 10\ \mathrm{f}\mathrm{o}\mathrm{r}\ \mathrm{medaka}\kern0.5em \mathrm{AND}\kern0.5em \mathrm{r}\mathrm{ead}\ \mathrm{coun}{\mathrm{t}}_{\mathrm{t}\mathrm{estis}} > 50 $$

$$ \mathrm{sum}{\left(\mathrm{read}\ \mathrm{coun}\mathrm{t}\right)}_{\mathrm{somatic}} < 6\ \mathrm{f}\mathrm{o}\mathrm{r}\ \mathrm{platyfish}\kern0.5em \mathrm{AND}\kern0.5em \mathrm{r}\mathrm{ead}\ \mathrm{coun}{\mathrm{t}}_{\mathrm{t}\mathrm{estis}} > 50 $$

and a second set with samples that are expressed in both testis and somatic cells, where: testes and at least one somatic sample is required to be expressed (read count > =10).

### Further characterization of the piRNA reference data

To assess sequence similarity between Xiphophorus and medaka, references were mapped to the reference sequences of the other species. Subsequently, the final reference sets of both fish were blasted against known transposable elements (TEs) of each fish (in-house collection, also used in Chalopin et al. [17]). To examine whether the reference sequences show a bias towards uridine in the first position and towards adenine at the 10th position as stated in Kawaoka et al. [32] we calculated the base distribution for sequences that have a hit within a reference cluster.

To decide whether piRNA sequences are located within a transcript region, sequences mapping to the reference were blasted against the respective transcriptome (identity > 97.5 %). For functional clustering the human homologues were determined using Ensembl biomart with default settings. Transcripts with *Blast* hits in both medaka and *Xiphophorus* or in both fish and human piRNA clusters were functionally clustered using the tools DAVID (http://david.abcc.ncifcrf.gov/tools.jsp) and the ‘WEB-based GEne SeT AnaLysis Toolkit’ webgestalt (http://bioinfo.vanderbilt.edu/webgestalt/option.php).

### Comparison with human piRNAs

For the comparison of fish and human piRNA clusters human piRNA sequences from piRNA bank (http://pirnabank.ibab.ac.in) were downloaded and merged similar to the treatment of the fish piRNA sequences with a spacing of 1 kb. For sequence comparison between fish and human piRNA clusters Blast was used (%identity > 90 and score > 1000). To compare genes within a distance of 1 kb human piRNAs were mapped to the genome and proximity to genes was calculated using closestBed.

### Detection of putative piRNA targets

For the detection of putative piRNA targets RNAhybrid (http://bibiserv.techfak.uni-bielefeld.de/rnahybrid#) was used. This is a tool for finding the minimum free energy hybridization of a long and a short RNA, primarily developed as a means for microRNA target prediction [57]. Thresholds for target selection were minimum free energy < −30 AND *p*-value < 0.05. The respective transcriptome was used as reference.

### Detection of differentially expressed piRNA clusters/transcripts

Differential expression of piRNA clusters or transcripts between tumor and benign control was calculated using the Bioconductor package DESeq2 [58] based on the number of reads mapping to each cluster or reads for each transcript. Fold regulation for either cluster or mRNA was considered only, if at least one group in a comparison had a read count > 10 and logFC > 2.

### Reverse transcription and real-time PCR for piRNAs and mRNAs

To confirm the expression of piRNAs on a larger number of tumors by qPCR we selected 3 piRNA clusters that were expressed in tumors and had a log2FC (IM vs. HP) > 2. Within this region we chose the most highly expressed sequence parts as shown in Additional file 9: Figure S6. Briefly, 200–500 ng of small RNA was polyuridylated by poly(U) polymerase (NEB, Germany) at 37 °C for 30 min in 20 μl reaction volume in 1x M-MuLv RT buffer supplemented with RNase inhibitor and 0.5 mM rUTP followed by addition of 10 μl RT-reaction mixture (0.5 μg SL-poly(A) primer, dNTPs, M-MuLV RT enzyme and buffer). The incubation was continued for 1 h at 37 °C and then terminated at 70 °C for 5 min. Real-time PCR was performed in 25 μl reactions with SYBR green containing reaction mixture for PCR, 3 ng of small-RNA-cDNA, universal primer and piRNA specific primer. All results are averages of 2 PCR experiments. Results were normalized to U6 SnRNA as 2^-Δ^Ct. Real-time PCR for mRNAs was performed in 25 μl reactions with SYBR green containing reaction mixture for PCR, 40 ng equivalent of RNA-cDNA and gene specific primers. Results were normalized to ef1a mRNA levels. Oligonucleotides used in this study are listed in Additional file 10: Table S4.

### Availability of supporting data

Additional file 11: Table S5: BED file of medaka piRNA clusters.

Additional file 12: Table S6: BED file of *Xiphophorus* piRNA clusters.

## Additional files

Additional file 1: Figure S1. Flow chart of the reference construction. (PDF 34 kb) Additional file 2: Table S1. Sample sequence distribution. The percentage of clean reads, as supplied by BGI, was between 98.73 and 99.67 % of total read number. On average 89.25 % of all clean reads were mapped to the genome. The number of sequences with a length between 25 and 32 bases was on average 3x higher in ovary and testis than in somatic tissues. In contrast to this, the proportion of sequences mapping to known miRNAs from mirBase or non-coding RNA annotated as miRNA from the ncrna dataset from Ensembl for *Xiphophorus* (Xiphophorus_maculatus.Xipmac4.4.2.ncrna.fa) was significantly higher in somatic cells than in germline cells. In oxidized samples a clear reduction of RNAs other than piRNAs could be observed. (XLSX 14 kb) Additional file 3: Figure S2. Length distribution of the sequences after oxidation for medaka and Xiphophorus. (PDF 667 kb) Additional file 4: Figure S3. Pie charts of the base distribution of piRNA sequences: medaka at the first position (A) and at the 10th position (B) and of Xiphophorus at sequence position 1 (C) and 10 (D). (PDF 310 kb) Additional file 5: Table S2. Number of sequences produced by the primary or secondary biogenesis pathway. First column: absolute numbers of sequence reads with uridine at position1 (1U) and no adenine at the 10th position (10A) indicating piRNAs generated by the primary pathway. 2nd column: sequence reads with no uridine at position 1 but 10A. All other sequence reads were excluded. Only sequence reads mapping to germline, tumor or control samples in medaka and Xiphophorus were considered. 3rd column: ratio for 1U/10A as a measure for the preference of the primary or secondary biogenesis pathway was calculated. Column 4–6: analogous to columns 1–3, but with guanine instead of uridine and adenine. (XLSX 11 kb) Additional file 6: Table S3. Target genes of four selected piRNAs as predicted by RNAhybrid. Thresholds for target selection were minimum free energy < −30 AND *p*-value < 0.05. In the 4th column log fold changes of the genes in the corresponding mRNA dataset are given. (XLSX 14 kb) Additional file 7: Figure S4. Directed acyclic graph (DAG) of enriched gene ontology (GO) categories derived from WebGestalt for genes located in both medaka and Xiphophorus piRNA clusters. To be selected as “common”, piRNA cluster sequences of both fish had to align by Blast with %identity > 90 and score > 1000. Categories labeled in red represent enriched categories. Categories labeled in black represent their non-enriched parents. (PNG 83 kb) Additional file 8: Figure S5. Venn diagram of numbers of genes mapping to a piRNA. To compare gene lists, human homologes of medaka and Xiphophorus were obtained from Ensembl biomaRt using default settings. (PNG 77 kb) Additional file 9: Figure S6. Histogram of bases within piRNA cluster 3. Red rectangles indicate regions selected for primer design. (PDF 42 kb) Additional file 10: Table S4. Primers used for quantitative PCR for piRNA and mRNA. (XLSX 11 kb) Additional file 11: Table S5. Bed file of medaka piRNA clusters. (BED 2235 kb) Additional file 12: Table S6. Bed file of *Xiphophorus* piRNA clusters. (BED 1072 kb)

## Abbreviations

ESC: embryonic stem cell
FM: Fin melanoma
HP: Hyperpigmented skin
IM: Invasive melanoma
Ol: Oryzias latipes, medaka
Piwil: P-element induced wimpy testis
TE: transposable element
UM: Uveal melanoma
XE: Exophytically growing xanthoerythrophoroma
Xhe: Xiphophorus hellerii
