# Supplementary material for: Germ cell and tumor associated piRNAs in the medaka and Xiphophorus melanoma models
Source: BMC Genomics. 2016 May 17;17:357. doi: 10.1186/s12864-016-2697-z (PMC4869193; doi:10.1186/s12864-016-2697-z)
Supplement: Additional file 1: Figure S1. — Flow chart of the reference construction. (PDF 34 kb) [file 12864_2016_2697_MOESM1_ESM.pdf]

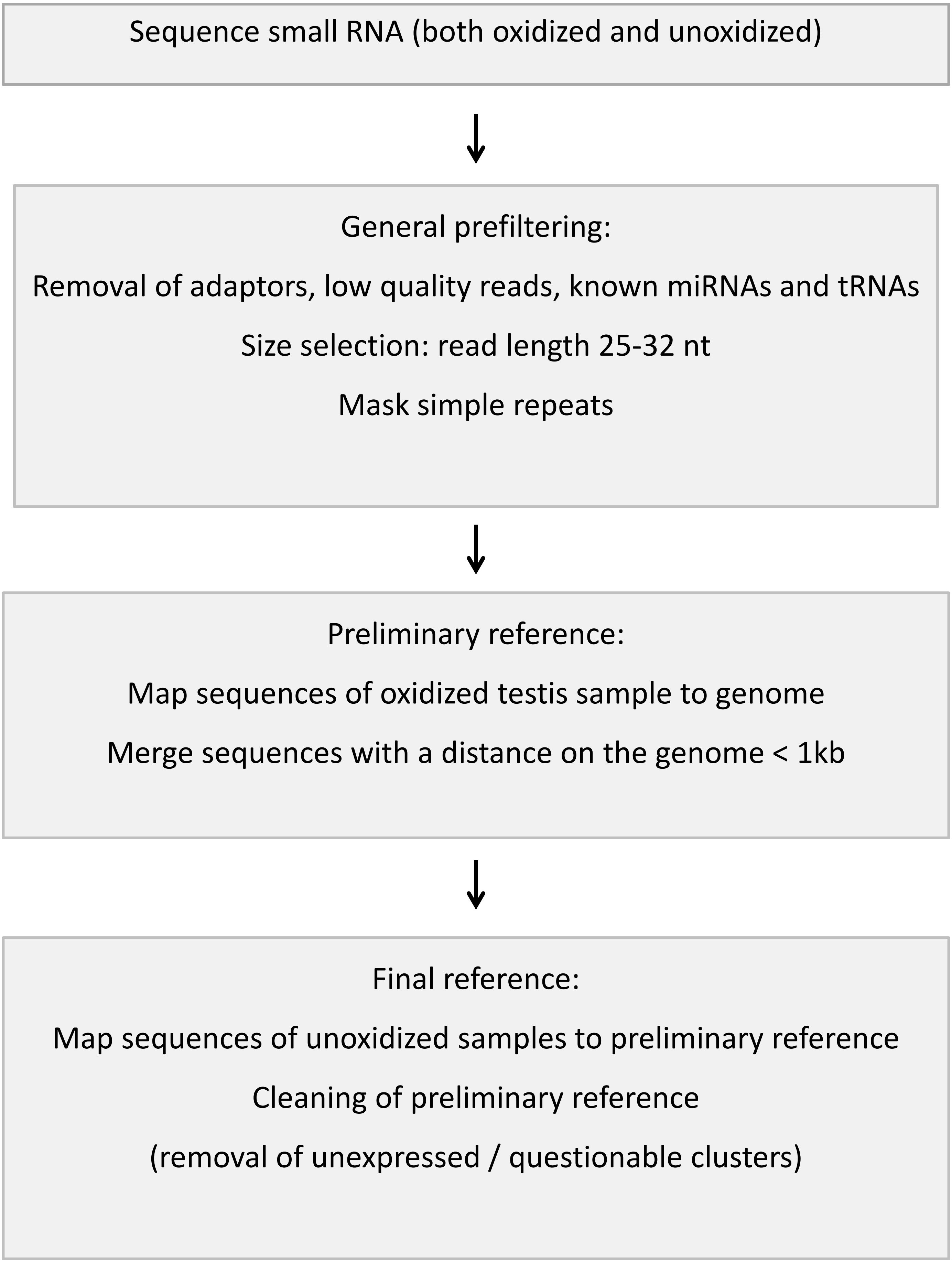
Sequence small RNA (both oxidized and unoxidized)
General prefiltering:
Removal of adaptors, low quality reads, known miRNAs and tRNAs
Size selection: read length 25-32 nt
Mask simple repeats
Preliminary reference:
Map sequences of oxidized testis sample to genome
Merge sequences with a distance on the genome < 1kb
Final reference:
Map sequences of unoxidized samples to preliminary reference
Cleaning of preliminary reference
(removal of unexpressed / questionable clusters)
