# Supplementary figures and images for: Germ cell and tumor associated piRNAs in the medaka and Xiphophorus melanoma models
Source: BMC Genomics. 2016 May 17;17:357. doi: 10.1186/s12864-016-2697-z (PMC4869193; doi:10.1186/s12864-016-2697-z)

## Medaka

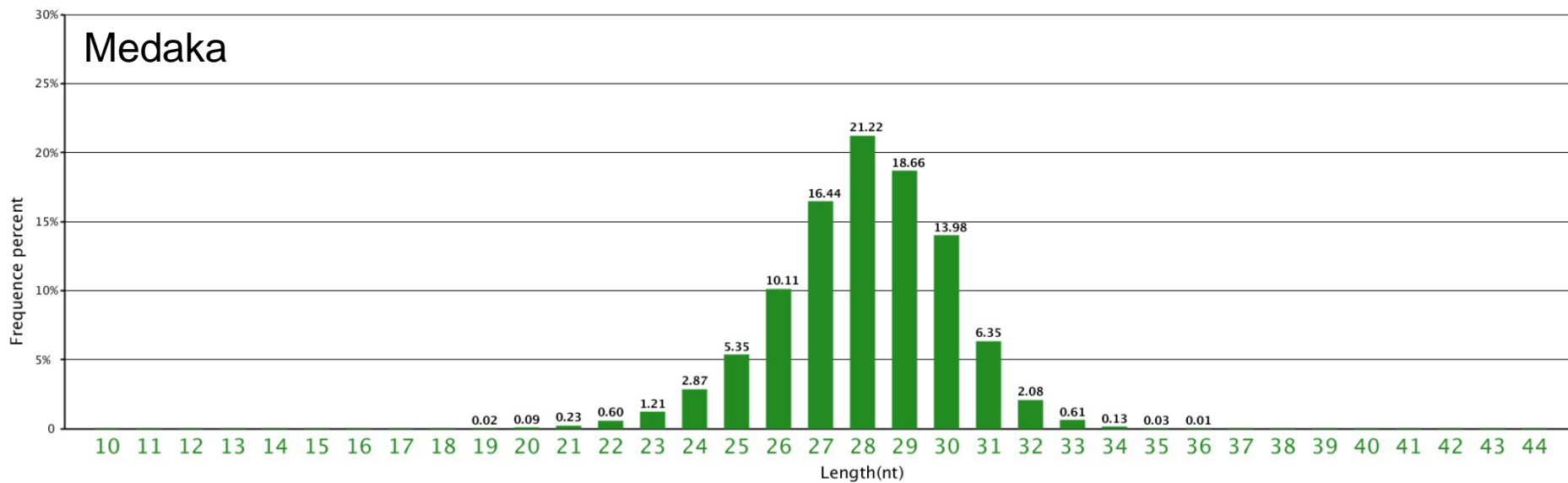

## Xiphophorus

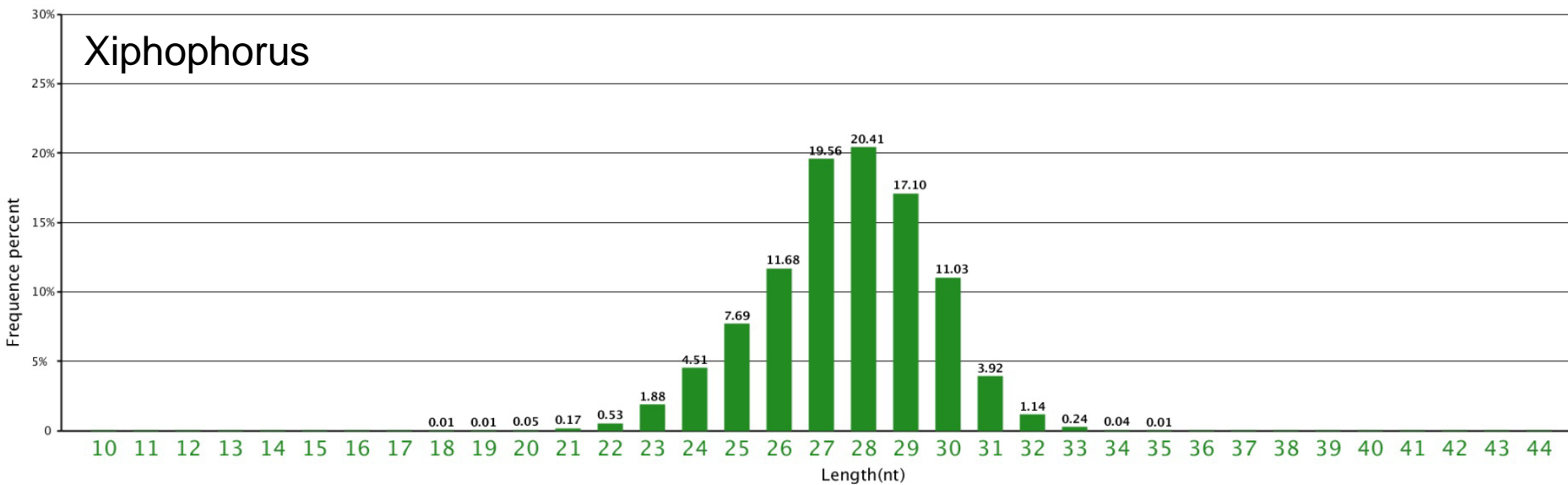

Supplement: Additional file 3: Figure S2. — Length distribution of the sequences after oxidation for medaka and Xiphophorus. (PDF 667 kb) [file 12864_2016_2697_MOESM3_ESM.pdf]

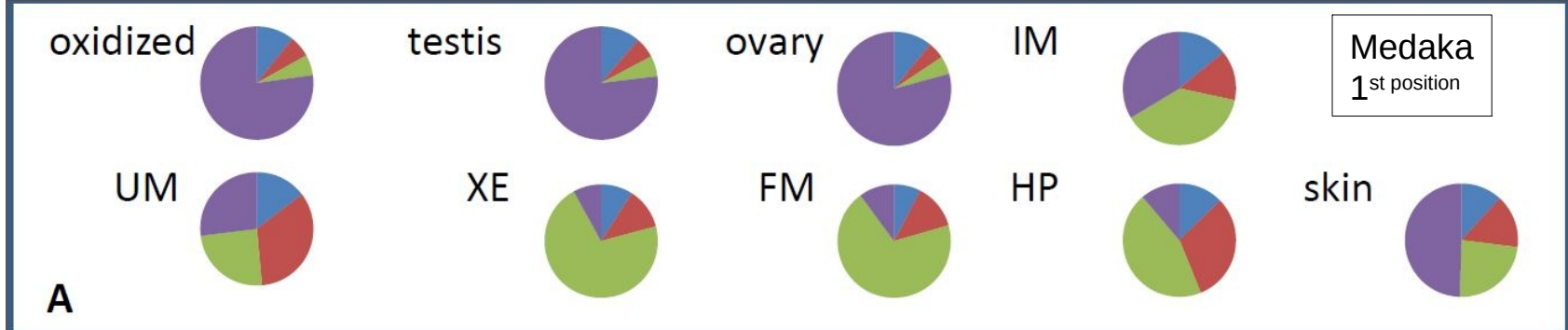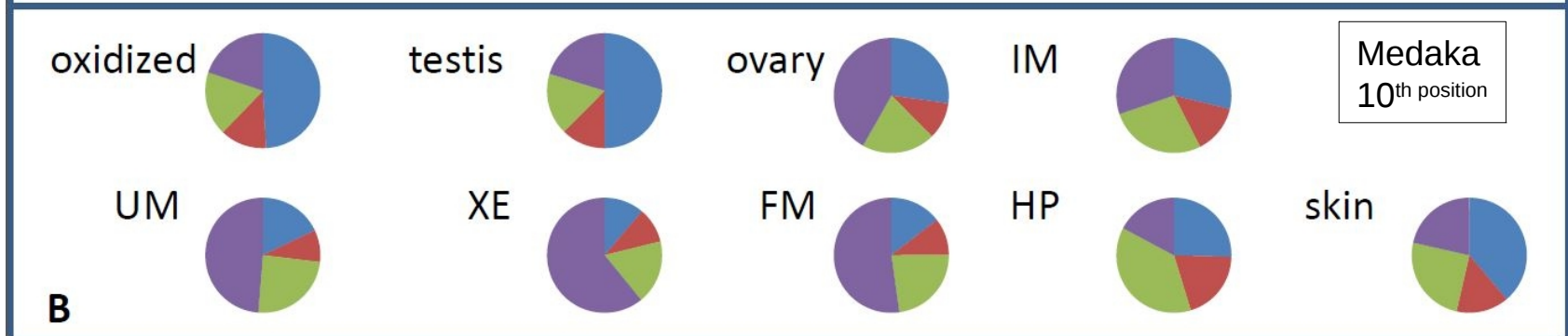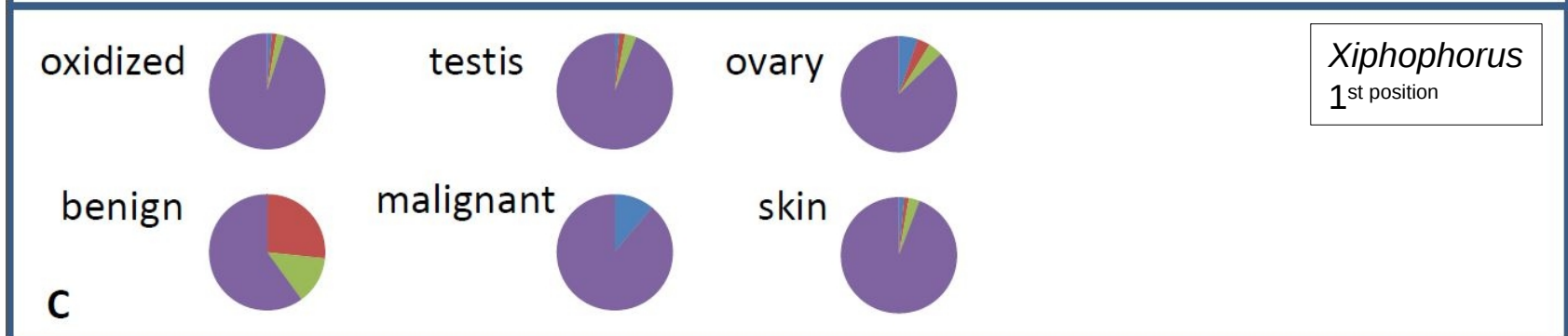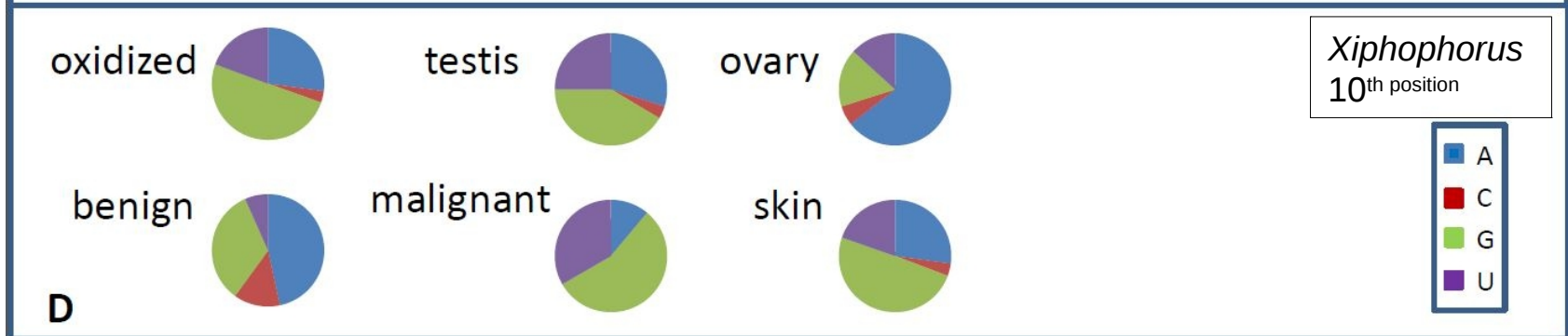

Supplement: Additional file 4: Figure S3. — Pie charts of the base distribution of piRNA sequences: medaka at the first position (A) and at the 10th position (B) and of Xiphophorus at sequence position 1 (C) and 10 (D). (PDF 310 kb) [file 12864_2016_2697_MOESM4_ESM.pdf]

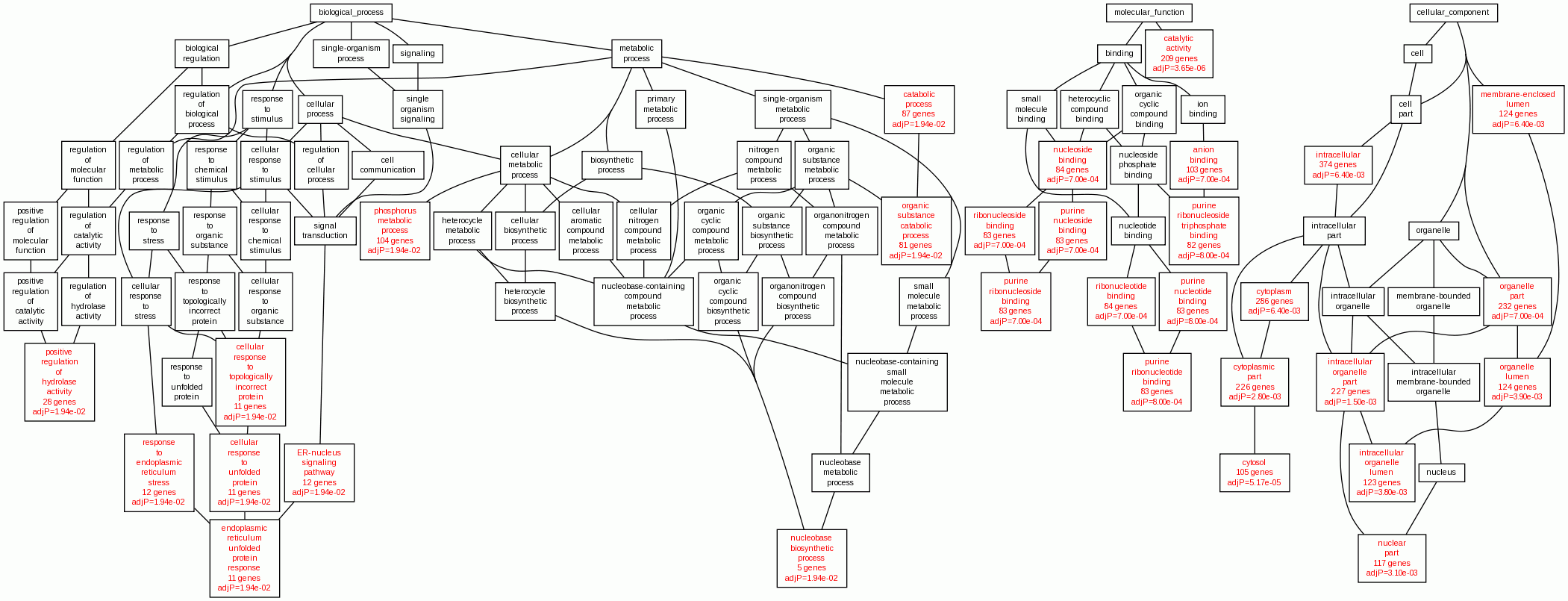

Supplement: Additional file 7: Figure S4. — Directed acyclic graph (DAG) of enriched gene ontology (GO) categories derived from WebGestalt for genes located in both medaka and Xiphophorus piRNA clusters. To be selected as “common”, piRNA cluster sequences of both fish had to align by Blast with %identity > 90 and score > 1000. Categories labeled in red represent enriched categories. Categories labeled in black represent their non-enriched parents. (PNG 83 kb) [file 12864_2016_2697_MOESM7_ESM.png]

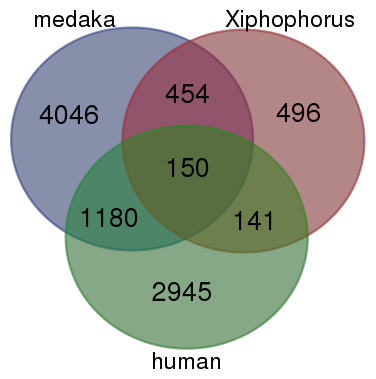

Supplement: Additional file 8: Figure S5. — Venn diagram of numbers of genes mapping to a piRNA. To compare gene lists, human homologes of medaka and Xiphophorus were obtained from Ensembl biomaRt using default settings. (PNG 77 kb) [file 12864_2016_2697_MOESM8_ESM.png]

reference

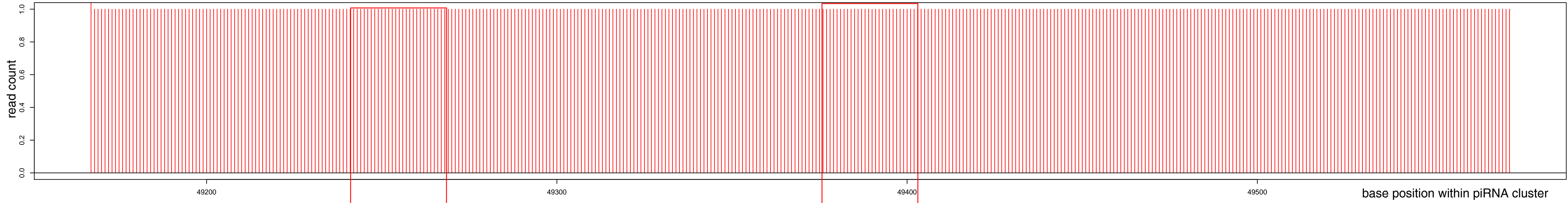

testes

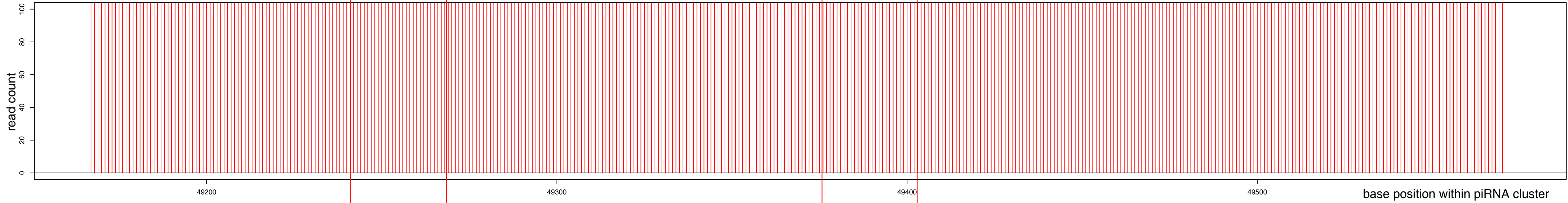

ovaries

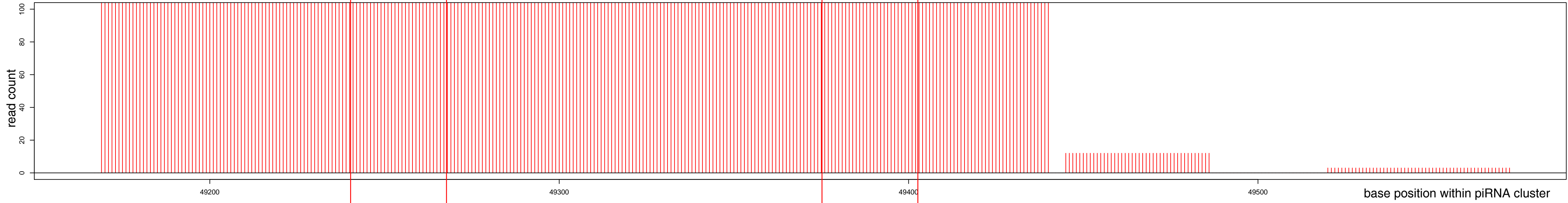

UM

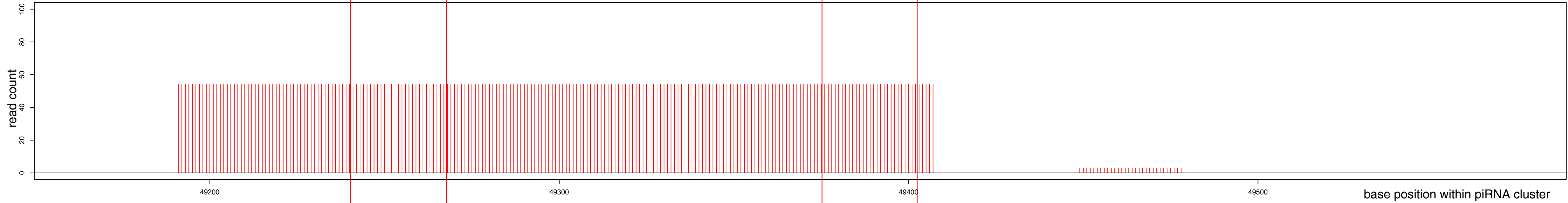

IM

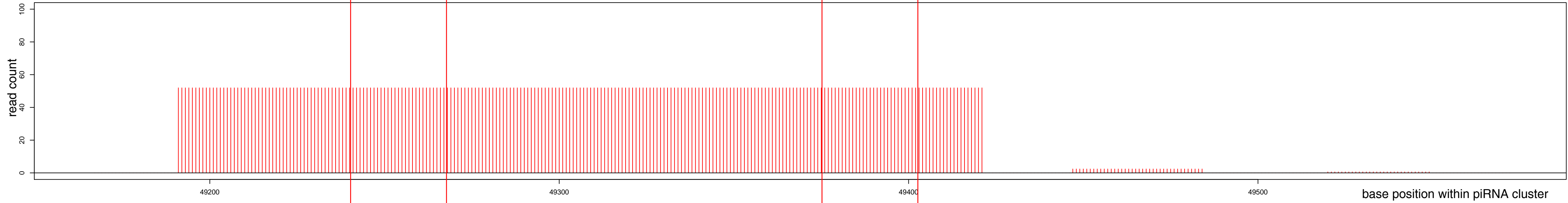

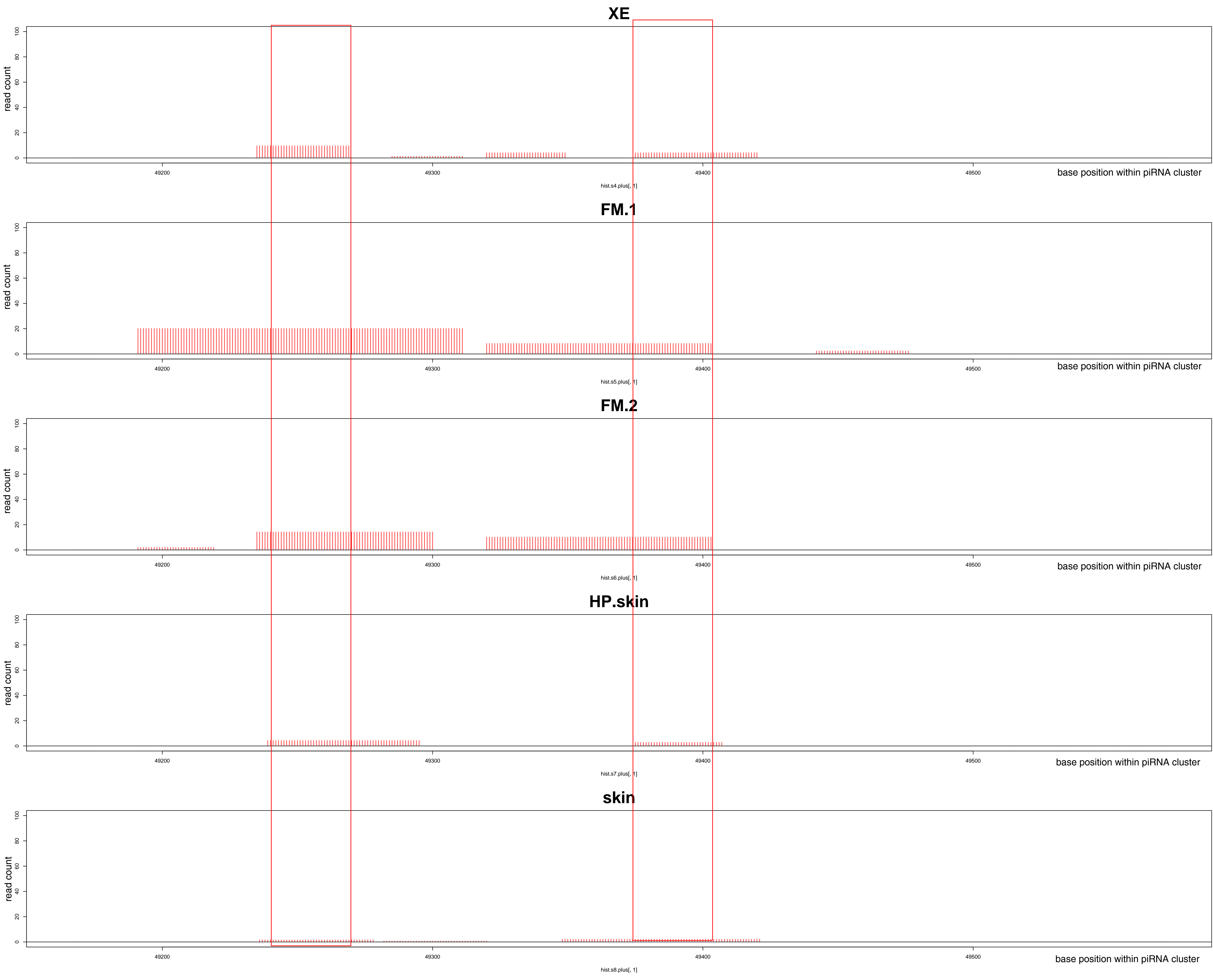

Supplement: Additional file 9: Figure S6. — Histogram of bases within piRNA cluster 3. Red rectangles indicate regions selected for primer design. (PDF 42 kb) [file 12864_2016_2697_MOESM9_ESM.pdf]
